# Collaborative Development of Feedback Concept Maps for Virtual Patient–Based Clinical Reasoning Education: Mixed Methods Study

**DOI:** 10.2196/57331

**Published:** 2025-01-30

**Authors:** Anja Mayer, Inga Hege, Andrzej A Kononowicz, Anja Müller, Małgorzata Sudacka

**Affiliations:** 1 Medical Education Sciences, University of Augsburg, Augsburg, Germany; 2 Institute for Research in Health Science Education, Brandenburg Medical School Theodor Fontane, Neuruppin, Germany; 3 Department of Bioinformatics and Telemedicine, Jagiellonian University Medical College, Kraków, Poland; 4 Department of Medical Education, Center for Innovative Medical Education, Jagiellonian University Medical College, Kraków, Poland

**Keywords:** clinical reasoning, consensus building process, concept map, consensus map, virtual patient, international collaboration, health professionals' education, undergraduate, collaborative, development, feedback, content analysis, health professional, medical student, mixed method, Europe, questionnaire, descriptive analysis

## Abstract

**Background:**

Concept maps are a suitable method for teaching clinical reasoning (CR). For example, in a concept map, findings, tests, differential diagnoses, and treatment options can be documented and connected to each other. When combined with virtual patients, automated feedback can be provided to the students’ concept maps. However, as CR is a nonlinear process, feedback concept maps that are created together by several individuals might address this issue and cover perspectives from different health professionals.

**Objective:**

In this study, we aimed to develop a collaborative process for creating feedback concept maps in virtual patient–based CR education.

**Methods:**

Health professionals of different specialties, nationalities, and levels of experience in education individually created concept maps and afterward reached a consensus on them in structured workshops. Then, medical students discussed the health professionals’ concept maps in focus groups. We performed a qualitative content analysis of the transcribed audio records and field notes and a descriptive comparison of the produced concept maps.

**Results:**

A total of 14 health professionals participated in 4 workshops, each with 3‐4 participants. In each workshop, they reached a consensus on 1 concept map, after discussing content and presentation, as well as rationales, and next steps. Overall, the structure of the workshops was well-received. The comparison of the produced concept maps showed that they varied widely in their scope and content. Consensus concept maps tended to contain more nodes and connections than individual ones. A total of 9 medical students participated in 2 focus groups of 4 and 5 participants. Their opinions on the concept maps’ features varied widely, balancing between the wish for an in-depth explanation and the flexibility of CR.

**Conclusions:**

Although the number of participating health professionals and students was relatively low, we were able to show that consensus workshops are a constructive method to create feedback concept maps that include different perspectives of health professionals with content that is useful to and accepted by students. Further research is needed to determine which features of feedback concept maps are most likely to improve learner outcomes and how to facilitate their construction in collaborative consensus workshops.

## Introduction

### Background

“Clinical reasoning encompasses health professionals thinking and acting in assessment, diagnostic, and management processes in clinical situations taking into account the patient’s specific circumstances and preferences” [1]. It is evident that health professionals in different disciplines (eg, physicians and nurses) differ in their reasoning approaches [2], and there are differences between novices and experts [3]. Even experienced health professionals of the same discipline do not follow the same diagnostic process, even when they are confronted with the same medical case, and ultimately arrive at the same diagnosis [4,5]. A study by Charlin et al [6] showed that experts’ case solutions also varied depending on the situation, for example, whether they were asked to give answers as an examinee or as a panel member.

The variety and nonlinearity of possible clinical reasoning (CR) approaches make CR training and assessment a highly complex matter [4,7]. Therefore, concept maps have been suggested as a useful method for training the CR skills of medical students [4,8], especially in terms of problem representation [9].

Concept mapping is a method used to represent concepts and their relationships in a visual diagram, using explanatory terms to relate concepts to each other [10]. A typical use in health education is to present students with a case scenario and have them create a concept map to represent their thought process as the case unfolds [9,11]. They can record relevant findings, tests, differential diagnoses, and treatment options and connect concepts to each other to visualize their CR process [8]. Torre et al [12] show that concept maps promote the connection between theory and practice and facilitate knowledge integration and critical thinking.

Teachers can ask students to create concept maps in different forms, depending on the purpose, such as freely from scratch or in a preconstructed form [10,11]. Because creating a comprehensive and accurate concept map is time-consuming and students need some time to learn how to do it, Daley and Torre [8] suggest the use of semistructured concept maps.

Concept maps have also been found to be suitable for measuring learning outcomes [13], and various ways of assessing and scoring concept maps, both qualitatively and quantitatively, have been described in the literature [14-17]. A study by Morse and Jutras [18] showed that working with concept maps had an effect on the students’ problem-solving performance only when feedback was provided. However, in order to provide students with feedback on their concept map, some form of “expert concept map” is needed to compare students’ results with [19], which can then be provided in real time in digital environments. Such “expert concept maps” can be created by a single teacher or by a panel of professionals or experts [19-21]. In their systematic review of different methods for assessing CR skills, Daniel et al [9] concluded that “using written cases, expert consensus is the most prevalent method” used to create concept maps as feedback for students. However, little is known about the process and challenges involved when health professionals are asked to reach a consensus on a concept map for teaching CR.

Recent studies suggest that virtual patients (VPs) are an appropriate method for training CR [22-24], especially for some components of this process, such as collecting data, generating differential diagnoses, or developing a treatment plan [25,26]. VPs are computer-based patient case scenarios that students can interact with [27]. Often, such scenarios are designed so that the cases gradually lead the student to the final diagnosis by providing more and more information over time [28,29]. VPs provide a safe environment, in which mistakes can be made without harming real patients [30]. It has been suggested that combining concept map activities with VPs can reinforce the educational effect of VPs in CR outcomes [31]. The importance of VPs has increased over the years [32], especially since the beginning of the COVID-19 pandemic, when direct patient contact and opportunities for CR training were limited [33].

### Objectives

In this study, we aimed to develop a collaborative process for creating feedback concept maps in VP-based CR education. From this, we derive the following research questions: (1) What are the similarities and differences of concept maps for teaching CR that have been created by individual health professionals and groups? (2) What themes emerge when health professionals are asked to jointly create a concept map in a consensus workshop? (3) What are the challenges and benefits of such consensus workshops? (4) What aspects of the consensus concept maps do medical students find helpful in learning CR?

## Methods

### Study Design

This study followed a convergent mixed methods approach. First, we asked health professionals from different disciplines to individually create concept maps for 2 VPs that would serve as feedback for medical students. We then conducted structured digital workshops for those health professionals in which they reached a consensus on the concept maps. After the workshops were finished, we conducted focus groups with medical students to discuss which aspects of the professionals’ concept maps they found helpful for learning CR.

### Ethical Considerations

The study was approved by the institutional review board of the Ludwig-Maximilians-University, Munich, Germany (21‐0941), and adhered to ethical guidelines. Informed consent was obtained from all participants prior to their participation in the study, with assurances of anonymity and confidentiality. Participants were informed of the objectives of the study and how the data collected would be used. In addition, strict measures were taken to protect the privacy and confidentiality of the study data. Students who participated in the focus groups received a US $16 voucher as compensation for their time.

### Data Collection

Between November 2021 and January 2022, we sent out emails to invite health professionals of different specialties, nationalities, and levels of experience in medical education to participate in our study. The email included study information and a written informed consent form. After returning the signed written consent by email, participants were asked to create concept maps for 2 VP cases. We used the software platform CASUS (Instruct gGmbH), which is a VP player and authoring environment with integrated concept map functionality [31]. The study participants were told that these concept maps would serve as feedback for medical students. We carefully chose the VPs with regard to their sociodemographic features, key symptoms, and difficulty levels. They were a 19-year-old female student with mononucleosis and a 58-year-old male nurse with hepatitis E. We chose them for providing patients of different sex, age, and profession. Both were heterosexual and of Caucasian origin. We deliberately chose these VPs because they provided different levels of difficulty for the students but had key symptoms that are common in daily practice and easily recognizable by health professionals of different specialties. The 2 VPs can be found on the CASUS platform and are part of a collection of over 200 freely available VPs in 6 languages [34]. These VPs include a semistructured concept map that students fill out while solving the case, covering 4 categories: findings, differential diagnoses, tests or examinations, and treatments [31].

**Figure 1.**
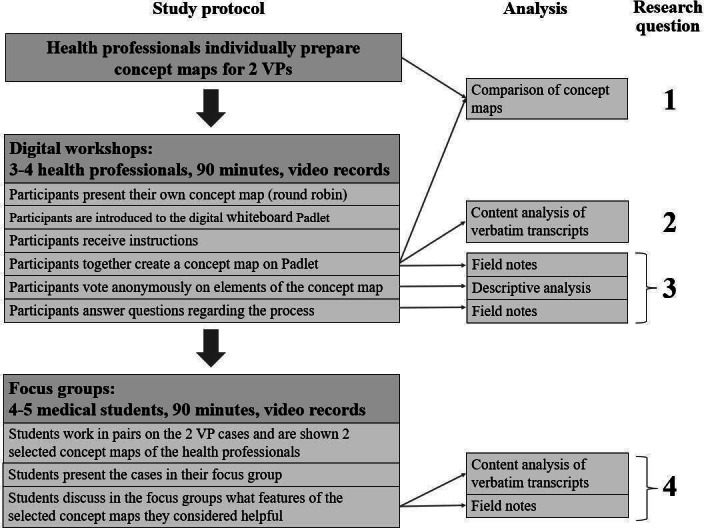
Flowchart of study protocol and analysis plan with regard to the research questions. VP: virtual patient.

Participants were also asked to complete a web-based questionnaire that included personal data and level of experience in concept mapping and teaching CR. We used a convenience sampling strategy, inviting partners from 2 recent Erasmus+ projects, iCoViP (International Collection of Virtual Patients) and DID-ACT (Developing, Implementing, and Disseminating an Adaptive Clinical Reasoning Curriculum for Healthcare Students and Educators) [35,36], who were interested in teaching CR with concept maps. As the VPs are available in multiple languages, we valued the international composition of the study group that would reduce local bias in clinical practice. Participants were given 10 days to create the individual concept maps and were reminded of the task 3 days before the workshop. After that, we held structured digital workshops of 90 minutes, where they met in groups of 3‐4 to reach a consensus on a common concept map. The workshops took place on the Zoom platform (Zoom Video Communications) and were video recorded. A Mayer and MS facilitated the workshops, following a predefined structure according to the nominal group technique [37,38] (Figure 1): first, all participants explained their own concept maps to the others in a round robin and described their reasoning. Then, A Mayer and MS introduced them to the digital whiteboard Padlet (Wallwisher Inc), and the participants had the opportunity to try it out. Once they felt comfortable with the tool, A Mayer and MS gave them instructions on how to create a concept map together. They then created a new concept map on Padlet based on their individual concept maps. A Mayer and MS answered participants’ questions, kept track of the timeline, and reminded participants of the original assignment if they strayed from the topic. When the concept map was complete, A Mayer and MS provided the opportunity to anonymously rate the concepts and connections with a thumbs up or down mechanism on Padlet. Afterward, they asked the participants about their experience of creating the concept map together. IH and AAK attended the workshops as neutral observers and, together with A Mayer and MS, took field notes, which they all discussed immediately after the workshop. The study was piloted as a face-to-face workshop in October 2021. Afterward, we decided that web-based meetings would be equally feasible and made minor changes to the study protocol, such as adding an anonymous voting round.

After all workshops were completed, IH and MS selected 4 individual and 4 consensus concept maps to be discussed by medical students in focus groups. For this purpose, 9 international medical students were recruited to participate in 90-minute focus groups during a transnational meeting of the iCoViP project. Written informed consent was obtained prior to participation. In the beginning, the students were asked to work in small teams (2‐3 students) and solve 1 of the 2 VP cases together. Afterward, the teams were shown 2 of the selected concept maps from the workshops to compare and decide which one they would prefer to have as feedback for their case and why. Then, 2 teams of students who had worked on different cases were brought together as a focus group. They presented their cases to the others and then started a group discussion, facilitated by A Mayer and MS, about which of the presented concept maps they found most helpful and how different features of the concept maps could improve their CR process.

### Data Analysis

This study used a convergent mixed methods design. In the quantitative part, a descriptive statistical analysis of the questionnaire, concept maps, and votes was performed using Microsoft Excel. The individual and consensus concept maps were analyzed for scope (number of nodes and connections), agreement (number of “likes” of nodes or connections in the consensus phases of the group concept map authors), and content (number of times a particular concept, eg, “fever,” appeared in the individual and consensus concept maps). We extracted information from the concept maps and compared them separately for each of the 2 cases.

The qualitative part of the study involved the thematic analysis of the transcripts and field notes from the workshops and focus groups. It was conducted in several steps. The recordings of the workshops were transcribed verbatim and anonymized. Two authors (A Mayer and A Müller) performed a thematic analysis of the transcripts, following the 6 steps for qualitative content analysis proposed by Kuckartz [39]. Using an inductive approach, they independently created codes for the first 2 workshops and reached a consensus on an initial coding framework. They then coded 1 workshop at a time, applying and refining the coding framework in an iterative process. They used MAXQDA software (version Analytics Pro 2022; VERBI GmbH) for coding and discussed discrepancies until a consensus was reached. A Mayer, A Müller, and IH then grouped similar codes into themes. Throughout the process, AAK and MS reviewed the coding framework and emerging themes and provided feedback; discrepancies were discussed until a consensus was reached.

We analyzed field notes taken during the workshops and participants’ responses during the round of questions for challenges and benefits. Student focus group recordings were transcribed verbatim and anonymized. Two authors (A Mayer and A Müller) independently extracted statements from the transcripts about what students found helpful in the selected concept maps, grouped them into themes, and discussed discrepancies until a consensus was reached. Finally, we looked for confirmation or discrepancies of the results obtained from the mixed methods.

## Results

### Participants

A total of 14 health professionals from 6 European countries participated in our study, of whom 9 were female and 5 were male. On average, participants were 37 (SD 10) years of age and had 10 (SD 9) years of professional experience. Participants worked in different disciplines (Table 1) and had an average of 6 (SD 5) years of experience in health education. Participants differed only slightly in their teaching experience with concept maps or CR.

**Table 1.** Characteristics of participating health professionals (N=14).

Characteristics	Values
Age (years), mean (SD)	37 (10)
**Sex, n (%)**	
Female	9 (64)
Male	5 (36)
**Country (place of work), n (%)**	
France	1 (7)
Germany	3 (21)
Poland	2 (14)
Portugal	1 (7)
Spain	4 (29)
Sweden	3 (21)
**Specialty, n (%)**	
Internal medicine	4 (29)
Nursing	2 (14)
Biochemistry	2 (14)
Rheumatology	2 (14)
Family medicine	1 (7)
Neurology	1 (7)
Paramedic	1 (7)
Occupational medicine	1 (7)
**Professional experience of physicians (n=9), n (%)**	
Resident	6 (67)
Consultant	3 (33)
Working experience (years), mean (SD)	10 (9)
Experience in health teaching (years), mean (SD)	6 (5)
**Experience in teaching with concept maps, n (%)**	
None	9 (64)
Some	5 (36)
Much	0 (0)
**Experience in teaching clinical reasoning, n (%)**	
None	5 (36)
Some	9 (64)
Much	0 (0)

Participants created 13 individual concept maps prior to the workshops. We held 4 digital workshops with 3‐4 participants each, resulting in 4 consensus concept maps (2 hepatitis E and 2 mononucleosis). We also conducted 2 focus groups with 4 and 5 medical students, respectively. The students were in their final year of study (sixth year), with an average age of 24 (SD 0.5) years. In total, 8 students were female, and 1 was male. We chose students from Portugal (n=5) and Poland (n=4) because these countries represent educational systems from different parts of Europe.

### Research Question 1: Comparison of Individual and Consensus Concept Maps

The individual concept maps varied widely from each other regarding scope and content. We found most similarities in the final diagnoses and treatment options and only a few similarities regarding findings, differential diagnoses, and tests. The same was true when comparing the consensus concept maps.

**Figure 2.**
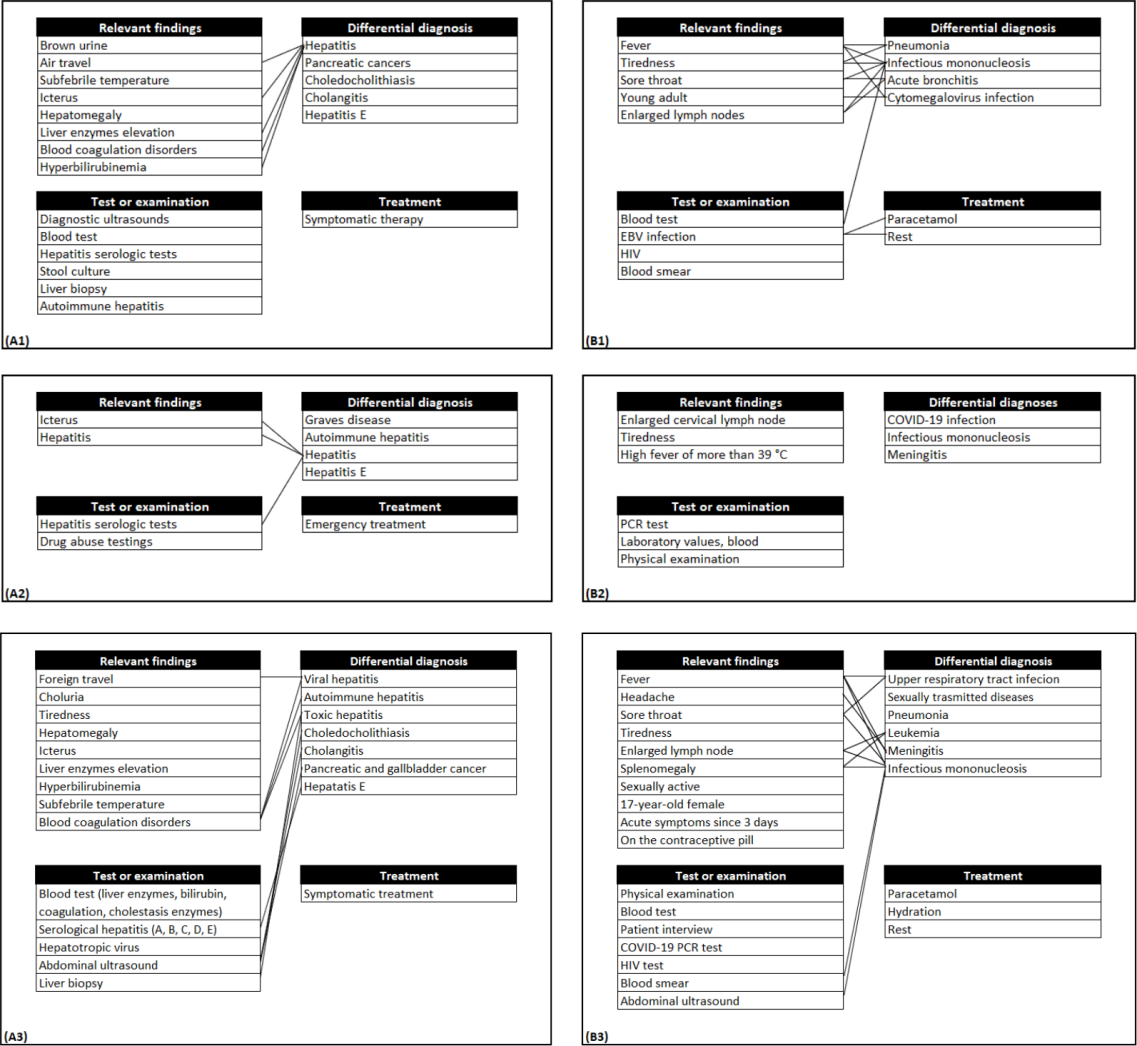
Examples of individual concept maps and consensus versions. (A1 and A2) Individual concept map (hepatitis E), (A3) consensus concept map (hepatitis E), (B1 and B2) individual concept map (mononucleosis), and (B3) consensus concept map (mononucleosis). EBV: Epstein-Barr virus; PCR: polymerase chain reaction

When we compared the consensus concept maps to the individual versions, we found that they all had a bigger scope than the individual concept maps, as can be seen in Table 2 and in the examples given in Figure 2 (original images are provided in Multimedia Appendix 1). We also found that most of the nodes from the individual concept maps were present in the consensus versions, and only in a few cases were nodes left out or new nodes added during the workshops. Altogether, the consensus versions showed higher similarities to the underlying individual versions than to each other. All consensus concept maps included connections, while these were missing in 5 of the individual versions.

**Table 2.** Comparison of number of elements in consensus and individual concept maps.

	Hepatitis E				Mononucleosis			
	Workshop 1		Workshop 2		Workshop 3		Workshop 4	
	n (GRP)^a^ (%)	Rn (IND)^b^	n (GRP) (%)	Rn (IND)	n (GRP) (%)	Rn (IND)	n (GRP) (%)	Rn (IND)
Total	56 (100)	10-29	33 (100)	11-25	39 (100)	13-22	43 (100)	9-11
**Nodes**								
Findings	9 (16)	6-8	9 (27)	1-8	10 (26)	3-6	12 (28)	3-4
Examinations or tests	11 (20)	2-8	5 (15)	2-5	7 (18)	2-4	7 (16)	0-3
Differential diagnoses	11 (20)	1-8	7 (21)	3-6	7 (18)	4-7	6 (14)	3-7
Treatments	1 (2)	1-1	1 (3)	1-1	3 (8)	0-2	2 (5)	0-0
Connections	24 (43)	0-13	11 (33)	3-5	12 (31)	0-13	16 (37)	0-0

a n (GRP): number of elements in the group consensus concept map.

b Rn (IND): range of element number in the individual concept maps.

### Research Question 2: Themes Emerging During the Consensus Process

From the qualitative content analysis of the creation phase, we identified 4 themes: the first theme covered the content of the consensus concept maps, that is, participants discussed which findings, examinations or tests, differential diagnoses, treatments, and connections should be included. Related to this, the second theme was the rationales they gave during their discussion, that is, why they thought something should (not) be part of the consensus concept map. The third theme covered the presentation of the consensus concept map, that is, participants discussed how to present the content. In the fourth theme, participants discussed their next steps, that is, how to approach the creation of the consensus concept map. Table 3 shows the 4 themes and associated subthemes, including sample quotes, and their frequency in the verbatim transcripts of the workshops.

**Table 3.** Themes and associated subthemes derived from the qualitative content analysis.

Themes and subthemes	Description	Sample quotes	Values, n (%)
**Content of the consensus concept maps (n=677)**			
Relevant findings	Which findings (not) to include in the concept map	“Sore throat” [Workshop 4]	227 (41)
Differential diagnoses	Which differentials (not) to include in the concept map	“Should we add EBV and CMV as a differential?” [Workshop 1]	200 (30)
Tests or examinations	Which tests or examinations (not) to include in the concept map	“Liver biopsy could be relevant” [Workshop 2]	170 (25)
Treatment	Which treatments (not) to include in the concept map	“Paracetamol I think is perfect in this case” [Workshop 3]	25 (4)
Connections	Which connections (not) to include in the concept map	“Maybe this splenomegaly should be also connected?“ [Workshop 3]	55 (8)
**Presentation of the content (n=89)**			
Layout	Visual aspects of the concept map or possible actions such as highlighting, crossing out, rearranging, merging, splitting, enlarging, or reducing nodes	“I’m just putting this in a nice order” [Workshop 3], “Can I change the color of the connections?” [Workshop 1]	40 (45)
Categorization	Which heading a node should be assigned to	“Could we add other headings, for example ‘recommendations’?” [Workshop 4]	6 (7)
Phrasing	Use of synonyms or abbreviations	“Is writing ‘STDs’ appropriate or should I use ‘sexually transmitted disease’?” [Workshop 3]	32 (36)
Level of granularity	Detail level of the concepts	“Viral hepatitis, autoimmune hepatitis or [just] hepatitis?” [Workshop 2]	11 (12)
**Rationales (n=318)**			
Medical relations	Medical relations between the concepts, including the probability of differential diagnoses	“I think about it because it’s a young female on the pill” [Workshop 3], “We think about it because it’s quite common” [Workshop 1]	96 (30)
Relevance	Highlighting the medical urgency or indicating that most participants are of the same opinion	“It’s a potentially dangerous situation for our patient” [Workshop 3], “And also COVID-19, I think we all agree” [Workshop 4]	42 (13)
Individual concept maps	Referring to individual concept maps or clinical reasoning process when creating them	“In the individual mapping, we have PCR-test, someone wrote that” [Workshop 4], “Was this something you came up with now during this process or [when creating your concept map]?” [Workshop 1]	62 (19)
Referring to the case	Referring to the case by quoting or repeating facts	“He’s not saying that he takes any drugs” [Workshop 2], “The case has provided us a biopsy” [Workshop 1]	65 (20)
Professional experience	What participants have experienced in daily practice or what they are accustomed to doing	“This is usually the first serology I order” [Workshop 1]	19 (6)
Common knowledge	General phenomena in society or “universal truths”	“People lie – he might be an alcoholic” [Workshop 2]“One would expect that this nurse is already immunized” [Workshop 1]	5 (2)
Encounter setting	Regional standards or differences between facilities (hospital, general practice, etc)	“How [do] you have it in Spain or Germany?” [Workshop 4], “I was wondering whether I would have done urine analysis in the [general] practice” [Workshop 1]	9 (3)
Hindsight	Assumption that the consensus process might be unconsciously guided by already knowing the final diagnosis	“[We think so because] we already know that it’s mononucleosis” [Workshop 3]	6 (2)
Didactical aspects	What could be helpful for the students or is the content appropriate for their level of knowledge, etc	“It could be a good training for students, to think what can cause hepatitis” [Workshop 1], “I think it’s too specialistic” [Workshop 3]	8 (3)
Functionality of the VP^a^ platform	Features, navigation, or structure of the CASUS platform	“I don’t know if this is possible on CASUS” [Workshop 2]	6 (2)
**Next steps (n=107)**			
Developing a strategy	How to approach the creation of the concept map	“[Let’s] do differentials first before adding anything to tests” [Workshop 3]	78 (73)
Referring to facilitators	Referring to instructions given by facilitators or directly asking them for advice	“[It depends on] what is wanted or what is expected” [Workshop 1]	29 (27)

a VP: virtual patient.

### Research Question 3: Challenges and Benefits of the Workshops

The results presented here are a summary of the field notes from the creation phase and the final round of questions, expanded by a descriptive analysis of the voting round. From a technical point of view, there were some problems due to the digital format, for example, weak network signal, low audio quality, or some participants feeling uncomfortable using Padlet for the first time. Since none of the participants were native English speakers, some struggled to find the right terms or misunderstood what others were saying due to a lack of vocabulary or the speaker’s accent.

Regarding the different disciplines, it seemed that participants who had worked in their specialty for many years were somewhat biased by their daily experiences and had difficulty seeing the cases from a student’s point of view. Some of the participants who were not physicians by training struggled to find the right diagnosis and expressed their uncertainty about certain medical terms or conditions. It was noticeable that topics such as didactic purpose, uncertainty (probability of differential diagnoses), or logical arrangement of nodes were hardly discussed.

All participants were cooperative and reached a consensus on the concept maps in an amicable manner. For about 10% (n=10) of the nodes, half (or more) of the participants abstained from voting or gave a thumbs down. We compared these nodes with the verbatim transcripts and found that for 6 nodes, there was no evidence in the discussion that any of the participants disagreed.

In the final round of questions, participants reported that creating a concept map with others was a complex task. On the other hand, participants found the group work helpful in stimulating their reflection and that it was constructive to create concept maps that included perspectives of different health professionals. In general, the structure of the workshops and the given timeline for the different parts were well-received. The round robin was seen as a useful introduction that helped them to understand the reasoning of other participants. Some participants mentioned that it was difficult to create a concept map for a case that they had not developed themselves or that they struggled with the fact that the case evolved over time, which made it more difficult to agree on a final version. Participants had mixed feelings regarding the usefulness of the consensus concept maps. While some were satisfied with the final concept maps and expected them to be helpful for students, others found the concept maps too messy or crowded in the end.

### Research Question 4: What Students Considered as Helpful

When the medical students were asked whether they preferred the individual or consensus concept maps, there was a slight tendency toward the consensus versions as they contained more findings, which the students found helpful for their own CR process.

Regarding the content and scope of the concept maps, there was agreement that there should not be too many connections between nodes, as this was seen as more confusing than helpful. However, the students expressed contradictory opinions regarding the nodes. While some preferred the concept maps with only the most relevant nodes, others preferred those with a wider scope, as these would contain “the most details that we also agreed on while we were solving the case.”

The same was true for the presentation of the content. Some students suggested having more layout features, such as “some type of colors” or dropdown functions, while others preferred a clear design and simple structure. Regarding the granularity of the nodes, some suggested that “the feedback map should be more [general.] To give us freedom” and should use broad terms such as “blood test.” Others said that in their medical school, they “can’t just say ‘do blood test,’ [but] must be very specific”; therefore, more specific terms would be helpful in the concept maps.

## Discussion

### Main Findings

In this study, we described the process of collaborative authoring of concept maps to serve as feedback in CR education using VPs. The participants regarded the collective process stimulating for reflection and helpful to understand the perspectives of the other health professional groups. We were able to find confirmation for this qualitative finding quantitatively by showing that the consensus concept maps contained more nodes and connections than the individual ones. This can also have negative aspects, as in the consensus workshops, participants tended to collect all nodes from the individual concept maps into the consensus version instead of selecting only the most relevant ones, paying little attention to didactic aspects. The structure of the workshops was well-received, participants appreciated working in interprofessional groups and easily reached a consensus, supporting their additions to the concept maps by high scoring of the concept map elements. However, there were some challenges, such as technical problems or participants being biased by their daily practice as specialists.

The final-year medical students in our focus groups preferred a variety of features of the concept maps, most of which were contradictory. As a result, it remains unclear which features can improve learners’ outcomes and whether consensus concept maps are more suitable for teaching CR than individual ones.

### Implications of the Findings

Our research suggests that there are a few approaches to help health professionals reach a consensus on a concept map. The procedure we used for the workshops served its purpose and was well-received by the participants. Thus, the results of this study can be seen as an important step toward establishing a sound consensus concept map protocol, informing about the benefits and challenges, and leading to the following recommendations for improving the process in the future:

1. Regarding the technical aspects of the workshops, we recommend that participants be given access to the digital whiteboard prior to the workshop so that those who wish to can familiarize themselves with the tool in advance.
2. Since didactic aspects played a minor role in the creation of the consensus concept maps, we recommend that an independent person with experience in didactics and concept mapping participates at the workshop. An alternative would be to prepare a pedagogical guide or checklist to be considered when developing concept maps for teaching CR. If such an opportunity arises, addressing the pedagogical aspects of concept map development would be a helpful element of faculty development courses on VP authoring.
3. When considering concept maps for VPs that address general CR skills in medicine, such as the one in the iCoViP project repository, workshops should preferably involve only internal medicine or family medicine physicians to avoid specialty bias. This would be different if the goal of the VPs was to achieve learning objectives for specialty or interprofessional education from the outset.

In terms of real-world implementation, we consider this study an important step in providing more diverse feedback to students working on CR concept maps in the context of VPs. This study contributed to this by showing that the concept maps created by consensus groups were more elaborate, both in terms of representing many viewpoints and in terms of the number of concepts and connections. However, this study also showed that the consensus groups should be more effectively encouraged to discuss the pedagogical aspects of the concept maps, such as how to adjust the complexity to the level of knowledge or cognitive load of the students.

### Limitations

Our study has several limitations. First, the number of concept maps underlying the quantitative analysis was limited, so that the corresponding results should be interpreted with caution.

Second, it is possible that the results of the workshop are not applicable to “real-world” situations, in which colleagues work together on a concept map without being observed. The participants in our workshops were very polite to each other and tended to avoid disagreements, probably because most of them did not know each other. On the other hand, we were able to include the perspectives of professionals from different disciplines.

Third, we had a limited number of students in the focus groups. Our data suggest that the effect of different features of concept maps on individual learning and preferences may vary considerably from student to student. Future research is needed to explore this in more depth.

Fourth, the sample size of VPs and workshop participants was limited, which might make our findings less generalizable. However, we did not see any new themes emerging in the subsequent workshops and focus groups, suggesting that the qualitative analysis had reached its saturation point.

### Comparison With Prior Work

There is a large body of literature on the so-called “group concept mapping” [14,40], including approaches to optimizing group compositions [41] or to identifying different cognitive styles [42]. However, to the best of our knowledge, most of these studies only include undergraduate students. Therefore, our study can be considered unique in proposing a novel approach to consensus concept mapping for health professionals.

The structure of the workshops, derived from the nominal group technique and adjusted to the needs of digital education, can be seen as a major strength. First, the round robin allows participants to gain insight into one another’s CR approach. Second, participants found the consensus creation of the concept maps useful and inspiring. Third, anonymous voting at the end facilitates the interpretation of the final concept map, as it gives the participants’ view on each individual element without the need to openly criticize someone else’s ideas.

When we compared the individual concept maps, we found a common tendency but also a great deal of variation, with most having only the final diagnosis and treatment in common. This is supported by the work of McGaghie et al [43,44], which shows the wide variety of approaches to a concept map of pulmonary physiology, even among experts in the same field. Therefore, the consensus process in our approach increased the universality of the feedback concept maps. Another positive aspect is that the process contributed to a rational increase in the number of connections in the concept maps. As previous research has shown, well-chosen connections are an important element of this form of knowledge representation, which is helpful in CR education [45].

We did not exclude from the study professionals without teaching experience, as we did not see clear evidence that this might be a limiting factor in creating meaningful concept maps. This is supported by a study by Charlin et al [46], who found that teaching and nonteaching physicians were similarly well suited to be part of the reference panel for concordance tests used to assess complexity and ambiguity in CR.

While some authors suggest the use of concept maps for CR assessment [47], most researchers in the field are ambivalent on this issue [8,9]. This is consistent with our findings, which suggest that the complexity and variety of the CR process make it very challenging to generate “expert concept maps” that can be used as a gold standard against which student versions can be compared.

Participants reported that they found the consensus workshops useful for reflecting on their individual concept maps and CR approach. Therefore, such workshops could also be a suitable tool for improving the concept map development of the individual participants. Further research is needed to determine the impact of the workshops on participants’ ability to develop concept maps.

Our study focused on the creation of concept maps for medical students. Future research should investigate how this can be applied to other professions, as a recent meta-analysis suggests that concept maps are also an appropriate method for improving critical thinking skills in nursing students [48].

### Conclusions

By providing feedback concept maps that illustrate the complexity and diversity of the CR process, we aim to support students in reflecting on their own thinking. The collaborative creation of concept maps for teaching CR is an opportunity to integrate different perspectives of health professionals and to account for individual differences in the reasoning process. In our study, we described a process for developing such collaborative concept maps and identified themes that emerged in workshops using this process. The resulting consensus concept maps tended to contain more nodes and connections than those created by individual health professionals and were well-received by students. We consider this study an important step in establishing a robust method for collaboratively creating effective concept maps in CR education. Future studies will focus on streamlining the process and identifying the most effective pedagogical features of feedback concept maps.

## Supplementary material

10.2196/57331 Multimedia Appendix 1 Original images of individual concept maps and consensus versions.

## Abbreviations

CR: clinical reasoning
DID-ACT: Developing, Implementing, and Disseminating an Adaptive Clinical Reasoning Curriculum for Healthcare Students and Educators
iCoViP: International Collection of Virtual Patients
VP: virtual patient

## References

[1] Huesmann L, Sudacka M, Durning SJ (2023). Clinical reasoning: what do nurses, physicians, and students reason about. J Interprof Care.

[2] Vreugdenhil J, Somra S, Ket H (2023). Reasoning like a doctor or like a nurse? A systematic integrative review. Front Med (Lausanne).

[3] Cuthbert L, duBoulay B, Teather D, Teather B, Sharples M, duBoulay G (1999). Expert/novice differences in diagnostic medical cognition - a review of the literature. https://users.sussex.ac.uk/~bend/papers/csrp508.pdf.

[4] Durning SJ, Lubarsky S, Torre D, Dory V, Holmboe E (2015). Considering “nonlinearity” across the continuum in medical education assessment: supporting theory, practice, and future research directions. J Contin Educ Health Prof.

[5] Grant J, Marsden P (1988). Primary knowledge, medical education and consultant expertise. Med Educ.

[6] Charlin B, Gagnon R, Pelletier J (2006). Assessment of clinical reasoning in the context of uncertainty: the effect of variability within the reference panel. Med Educ.

[7] Charlin B, Lubarsky S, Millette B (2012). Clinical reasoning processes: unravelling complexity through graphical representation. Med Educ.

[8] Daley BJ, Torre DM (2010). Concept maps in medical education: an analytical literature review. Med Educ.

[9] Daniel M, Rencic J, Durning SJ (2019). Clinical reasoning assessment methods: a scoping review and practical guidance. Acad Med.

[10] Novak J, Cañas A (2007). Theoretical origins of concept maps, how to construct them, and uses in education. Reflect Educ.

[11] Vink S, van Tartwijk J, Verloop N, Gosselink M, Driessen E, Bolk J (2016). The articulation of integration of clinical and basic sciences in concept maps: differences between experienced and resident groups. Adv Health Sci Educ Theory Pract.

[12] Torre DM, Daley B, Stark-Schweitzer T, Siddartha S, Petkova J, Ziebert M (2007). A qualitative evaluation of medical student learning with concept maps. Med Teach.

[13] Hay DB (2007). Using concept maps to measure deep, surface and non‐learning outcomes. Stud Higher Educ.

[14] Torre D, German D, Daley B, Taylor D (2023). Concept mapping: an aid to teaching and learning: AMEE Guide No. 157. Med Teach.

[15] Anohina A, Grundspenkis J Scoring concept maps.

[16] Ruiz-Primo MA, Schultz SE, Li M, Shavelson RJ (2001). Comparison of the reliability and validity of scores from two concept-mapping techniques. J Res Sci Teach.

[17] Kinchin IM, Hay DB, Adams A (2000). How a qualitative approach to concept map analysis can be used to aid learning by illustrating patterns of conceptual development. Educ Res.

[18] Morse D, Jutras F (2008). Implementing concept-based learning in a large undergraduate classroom. CBE Life Sci Educ.

[19] Torre DM, Durning SJ, Daley BJ (2013). Twelve tips for teaching with concept maps in medical education. Med Teach.

[20] Cutrer WB, Castro D, Roy KM, Turner TL (2011). Use of an expert concept map as an advance organizer to improve understanding of respiratory failure. Med Teach.

[21] McGaghie WC, McCrimmon DR, Mitchell G, Thompson JA, Ravitch MM (2000). Quantitative concept mapping in pulmonary physiology: comparison of student and faculty knowledge structures. Adv Physiol Educ.

[22] Plackett R, Kassianos AP, Kambouri M (2020). Online patient simulation training to improve clinical reasoning: a feasibility randomised controlled trial. BMC Med Educ.

[23] Dekhtyar M, Park YS, Kalinyak J (2021). Use of a structured approach and virtual simulation practice to improve diagnostic reasoning. Diagnosis (Berl).

[24] Watari T, Tokuda Y, Owada M, Onigata K (2020). The utility of virtual patient simulations for clinical reasoning education. Int J Environ Res Public Health.

[25] Plackett R, Kassianos AP, Timmis J, Sheringham J, Schartau P, Kambouri M (2021). Using virtual patients to explore the clinical reasoning skills of medical students: mixed methods study. J Med Internet Res.

[26] Botezatu M, Hult H, Fors UG (2010). Virtual patient simulation: what do students make of it? A focus group study. BMC Med Educ.

[27] Kononowicz AA, Woodham L, Georg C, Edelbring S (2018). Virtual patient simulations for health professional education. Cochrane Database Syst Rev.

[28] Hege I, Dietl A, Kiesewetter J, Schelling J, Kiesewetter I (2018). How to tell a patient’s story? Influence of the case narrative design on the clinical reasoning process in virtual patients. Med Teach.

[29] Kononowicz AA, Narracott AJ, Manini S (2014). A framework for different levels of integration of computational models into web-based virtual patients. J Med Internet Res.

[30] Edelbring S, Dastmalchi M, Hult H, Lundberg IE, Dahlgren LO (2011). Experiencing virtual patients in clinical learning: a phenomenological study. Adv Health Sci Educ.

[31] Hege I, Kononowicz AA, Adler M (2017). A clinical reasoning tool for virtual patients: design-based research study. JMIR Med Educ.

[32] Lang VJ, Kogan J, Berman N, Torre D (2013). The evolving role of online virtual patients in internal medicine clerkship education nationally. Acad Med.

[33] Hege I, Sudacka M, Kononowicz AA (2020). Adaptation of an international virtual patient collection to the COVID-19 pandemic. GMS J Med Educ.

[34] Mayer A, Da Silva Domingues V, Hege I (2022). Planning a collection of virtual patients to train clinical reasoning: a blueprint representative of the European population. Int J Environ Res Public Health.

[35] International Collection of Virtual Patients.

[36] (2022). Project results. DID-ACT.

[37] Humphrey-Murto S, Varpio L, Gonsalves C, Wood TJ (2017). Using consensus group methods such as Delphi and Nominal Group in medical education research. Med Teach.

[38] McMillan SS, King M, Tully MP (2016). How to use the Nominal Group and Delphi techniques. Int J Clin Pharm.

[39] Kuckartz U (2002). Qualitative Text Analysis: A Guide to Methods, Practice & Using Software.

[40] Rosas SR (2017). Group concept mapping methodology: toward an epistemology of group conceptualization, complexity, and emergence. Qual Quant.

[41] Kinchin I, Hay D (2005). Using concept maps to optimize the composition of collaborative student groups: a pilot study. J Adv Nurs.

[42] Stoyanov S, Jablokow K, Rosas SR, Wopereis IGJH, Kirschner PA (2017). Concept mapping—an effective method for identifying diversity and congruity in cognitive style. Eval Program Plann.

[43] McGaghie WC, Boerger RL, McCrimmon DR, Ravitch MM (1994). Agreement among medical experts about the structure of concepts in pulmonary physiology. Acad Med.

[44] McGaghie WC, McCrimmon DR, Mitchell G, Thompson JA (2004). Concept mapping in pulmonary physiology using pathfinder scaling. Adv Health Sci Educ Theory Pract.

[45] Kononowicz AA, Torre D, Górski S, Nowakowski M, Hege I (2023). The association between quality of connections and diagnostic accuracy in student-generated concept maps for clinical reasoning education with virtual patients. GMS J Med Educ.

[46] Charlin B, Gagnon R, Sauvé E, Coletti M (2007). Composition of the panel of reference for concordance tests: do teaching functions have an impact on examinees’ ranks and absolute scores?. Med Teach.

[47] Radwan A, Abdelnasser A, Elaraby S, Talaat W (2018). Correlation between concept maps and clinical reasoning for final year medical students at the faculty of medicine—Suez Canal University. QJM.

[48] Yue M, Zhang M, Zhang C, Jin C (2017). The effectiveness of concept mapping on development of critical thinking in nursing education: a systematic review and meta-analysis. Nurse Educ Today.

