# Supplementary figures and images for: Collaborative Development of Feedback Concept Maps for Virtual Patient–Based Clinical Reasoning Education: Mixed Methods Study
Source: JMIR Med Educ. 2025 Jan 30;11:e57331. doi: 10.2196/57331 (PMC11801104; doi:10.2196/57331)

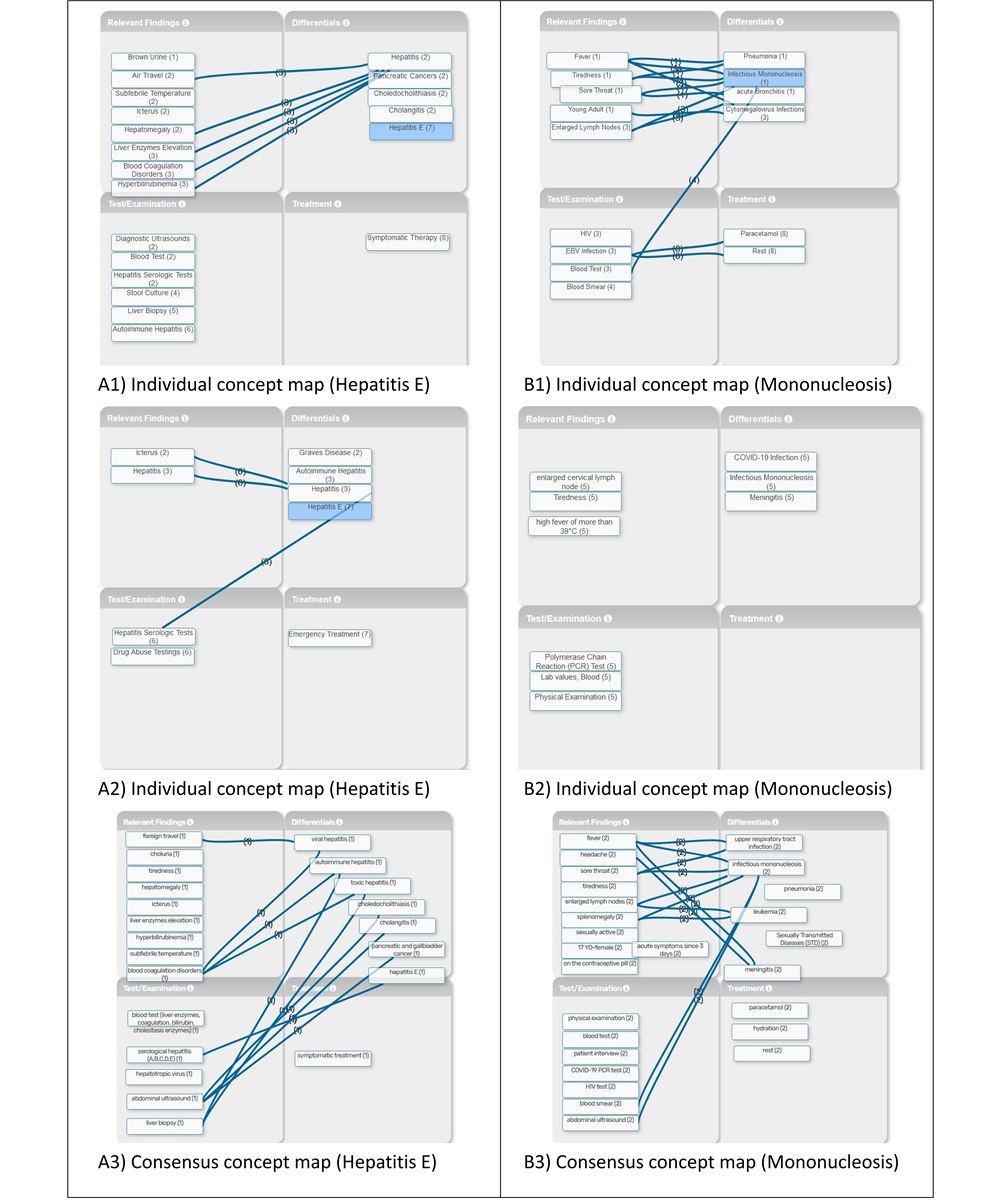

Supplement: Multimedia Appendix 1 [file mededu-v11-e57331-s001.png]
